# Histological Evaluation of the Effect of Local Application of *Punica granatum* Seed Oil on Bone Healing

**DOI:** 10.1155/2022/4266589

**Published:** 2022-09-24

**Authors:** Ibrahim Fouad Mohamed, Ban A. Ghani, Abdalbseet A. Fatalla

**Affiliations:** ^1^Department of Oral Diagnosis, College of Dentistry University of Baghdad, Baghdad, P. O. Box 1417, Iraq; ^2^Department of Prosthodontics, College of Dentistry University of Baghdad, Baghdad, P. O. Box 1417, Iraq

## Abstract

**Background:**

Bone healing is a complex and dynamic process that represents a well-orchestrated series of biological events of cellular recruitment, proliferation, and differentiation. The use of medicinal plants in bone healing has attracted increasing interest because of their lower side effects. *Punica granatum* seed oil (PSO) contains high levels of phenolic compounds, promotes osteoblast function, and plays an important role in bone remodeling. A gelatin sponge (Spongostan) is a hemostatic agent that is extensively applied as scaffolds in engineering and as drug carriers in the medical field. This study aimed to evaluate the effectiveness of PSO for bone healing enhancement. Twenty adult male New Zealand rabbits, weighing an average of 1.5–2 kg, were used in this study. Three intrabony holes were created in the tibiae of each animal, which were filled with a gelatin sponge (GS group) and combined gelatin sponge and PSO (GS/PSO group). Holes without material application were designated as the control group (C group). The animals were sacrificed at the healing duration (2–4 weeks) to prepare bone specimens for histological and histomorphometric analyses.

**Results:**

Histological findings indicated that the bone defects in the GS/PSO group showed more bone formation, mineralization, and maturation compared with the C and GS groups. Multiple group differences for bone cells showed a highly significant difference among all groups in the 2- and 4-week healing periods except for the C/GS and GS/GS/PSO groups at 4-weeks duration. Furthermore, highly significant results were obtained between both durations regarding the trabecular area, trabecular number, and bone marrow area.

**Conclusion:**

The study revealed that the combined application of GS and PSO was more effective in enhancing bone regeneration and accelerating bone healing compared with the other groups.

## 1. Introduction

The bone is a tissue organized into two main compartments, namely, trabecular bone (i.e., cancellous or spongy) and cortical bone (i.e., dense or compact) [1, 2]. Structurally, the bone is a nanocomposite compound composed of organic collagen nanofibers and inorganic compounds, such as hydroxyapatite and whitlockite [3].

Through the interactions of osteocytes (OC), osteoblasts (OB), and osteoclasts (OCL), bone tissue is characterized by a continually dynamic process of synthesis and breakdown of new bone and old tissue, respectively. As a result, when minor defects occur, bone tissue has an extraordinary ability for self-remodeling and self-healing [4, 5]. Researchers have focused on developing innovative therapeutic compounds that might promote bone repair and comprehending the inflammatory processes that regulate this process [6]. Besides their low costs and negligible side effects, the ability of natural bio-compounds to inhibit bone resorption and tissue inflammation while also increasing antioxidant defenses, tissue vascularization, and bone cell proliferation suggests that they may be a viable alternative for bone healing and regeneration [7].

Pomegranate (*Punica granatum*), a fruit native to the Middle East and India, has long been recognized as a rich source of flavonoids, vitamins, tannins, and immune-boosting antioxidants and has been empirically used for medicinal purposes [8].

The most significant therapeutically active polyphenols from pomegranates are ellagic and punicic acids, ellagitannins, anthocyanins, anthocyanidins, and isoflavones [9]. Gelatin sponge is derived from pig skin collagen and is widely used as a hemostatic agent. Due to its sterile, water-insoluble, malleable, biocompatible, biodegradable, noncarcinogenic, and nontoxic properties, it is extensively applied as engineering scaffolds and drug carriers in the medical field [10]. This study aims to histologically evaluate the effect of the topical application of *Punica granatum* seed oil (PSO) in promoting bone healing of induced bone defects in the rabbit tibia.

## 2. Materials and Methods

The materials used in this study are PSO (30 ml) containing pure cold-pressed seed extract (Mayan's Secret, USA, batch no. 1023820), absorbable gelatin sponge (Spongostan Dental, Ferrosan, Denmark, batch no. 63295), ketamine hydrochloride (50 mg), xylazine (2%), formalin (10%), ethanol alcohol (96%), xylol, paraffin wax, and hematoxylin and eosin (H&E) stain.

Twenty adult male New Zealand rabbits, weighing an average of 1.5–2 kg, were used in this study. Three intrabony holes (∼3 mm in diameter and 4 mm in depth) were created in the tibia of each animal. The rabbits were randomly divided into two groups for the 2- and 4-week healing periods, with 10 rabbits in each group. They were further subdivided into three groups: the control group (C), where no material was applied in the prepared hole; the GS group, where gelatin sponge (Spongostan) was applied to the hole; and the GS/PSO group, which received a combined application of Spongostan and PSO (10 *μ*l).

The animals were sacrificed to prepare bone specimens for histological and histomorphometric analyses of the bone microarchitectures (bone trabecular number [TN], trabecular area [TA], and bone marrow area [BMA]), and bone cell count (OB, OC, and OCL).

The experiment was conducted in accordance with the ethical principles for animal experiments of the College of Dentistry at the University of Baghdad (No. 424).

A general anesthetic solution was administered through intramuscular injection of 20 mg xylazine (0.2 ml/kg B. W.) and 50 mg ketamine HCL (20 mg/kg B. W.). Before the surgery, all surgical tools and surgical towels were sterilized in an autoclave at 121°C and a pressure of 15 bar/cm^2^ for 30 min. A skin incision was made on the underlying connective tissues, and tissue separation was carried out using a periosteal elevator to expose the medial aspect of the tibia. A vernier caliper was used to measure the desired depth, and a bur stopper was positioned on the surgical bur. Initial intermittent drilling was performed with a tiny round bur, and vigorous irrigation with normal saline was conducted using a 5 cc syringe. Thereafter, the region was thoroughly cleansed with sterile normal saline and dried with gauze. The hole in the right tibia was filled with PSO using a pipette and gelatin sponge. One of the two holes in the opposite tibia was cleansed only with normal saline to remove debris and left to heal freely as a control, whereas the second hole was filled with a gelatin sponge (Spongostan). All animals were scarified through an overdose with an anesthetic solution. A bone specimen was obtained by cutting the bone at least 5 mm away from the operation site with continuous saline irrigation. The specimens were immediately fixed in 10% freshly prepared formalin and left for 2 days for fixation. Bone decalcification was performed by using a formic acid-sodium citrate solution, which was freshly prepared from two solutions (125 cc of formic acid (90%) and 125 cc of distilled water, and 50 mg sodium citrate and 250 cc of distilled water) [11].

### 2.1. Histomorphometric Analysis of Bone Microarchitecture

Bone cells (OB, OC, and OCL) in histological sections stained with H&E at two different healing times (2- and 4-weeks) were counted, and the mean was obtained. Trabecular number (TN), trabecular area (TA), and bone marrow area (BMA) were measured by ImageJ, an image processing application created by the National Institutes of Health [12].

## 3. Results

**Figure 1:**
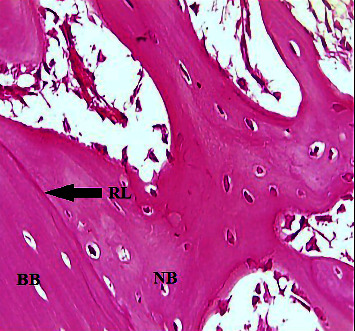
A view of the defect site in the C group shows a reversal line (RL), basal bone (BB), and new bone (NB) (arrow) after 2-weeks. H&E x40.

**Figure 2:**
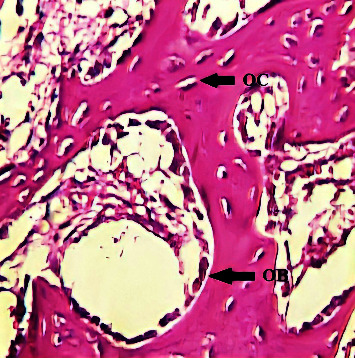
A view of the defect site in the GS group shows OB rimming newly formed trabeculae after 2-weeks. H&E x40.

**Figure 3:**
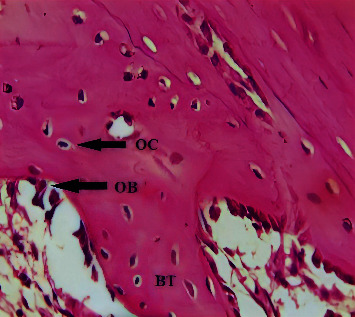
A view of the defect site in the GS/PSO group shows OB, OC, and bone trabeculae (BT) after 2-weeks. H&E x40.

A microscopical view of the defect area in the C group at 2-weeks showed newly formed bone trabeculae enclosing areas of marrow tissue and numerous OC embedded in the bone. A reversal line was observed separating old and new bone, and an OB rimming bone was noted (Figure 1). The GS group showed the OB rimming bone trabeculae with a large number of OC embedded in the bone (Figure 2). A view of the bone section of the GS/PSO group showed well-developed bone trabeculae at the defect area with OB at the periphery and OC embedded in the bone (Figure 3).

**Figure 4:**
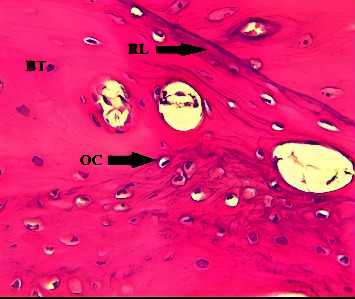
A view of the defect site in the C group shows numerous irregular OC trapped in bone trabeculae (BT) and reversal lines (RL) (arrows) after 4-weeks. H&E x40.

**Figure 5:**
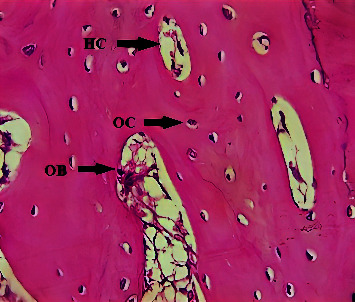
A view of the defect site in the GS group shows bone trabeculae surrounding the Haversian canals (HC), which are lined by OB and surrounded by OC, after 4-weeks. H&E x40.

**Figure 6:**
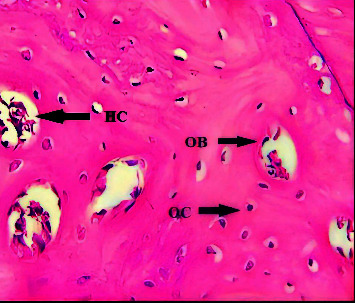
A view of the defect site in the GS/PSO group shows the Haversian canals lined by OB and surrounded by OC after 4-weeks. H&E x40.

After 4-weeks, the C group showed a dense mature bone almost filling the defect site, with OC arranged around the Haversian canal (Figure 4). The histological section of the GS group showed mature bone filling the defect site and Haversian canals surrounded by OC and rimmed by OB (Figure 5). The GS/PSO group showed mature, dense bone that coalesced with basal bone, with a few OC that were regularly arranged (Figure 6).

### 3.1. Histomorphometric Analysis of Bone Architecture Parameters

**Figure 7:**
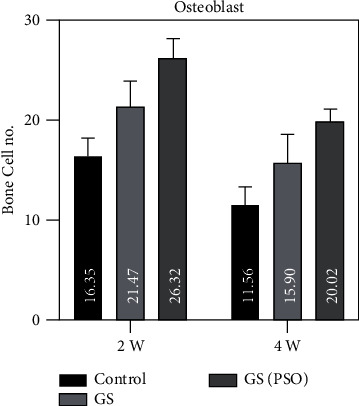
A bar chart representation of the mean and standard deviation of bone cells after 2- and 4-week time intervals for osteoblast, considering the C, GS, and GS/PSO groups.

**Figure 8:**
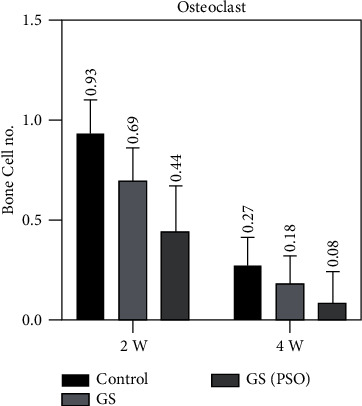
A bar chart representation of the mean and standard deviation of bone cells after 2- and 4-week time intervals for osteoclast, considering the C, GS, and GS/PSO groups.

**Figure 9:**
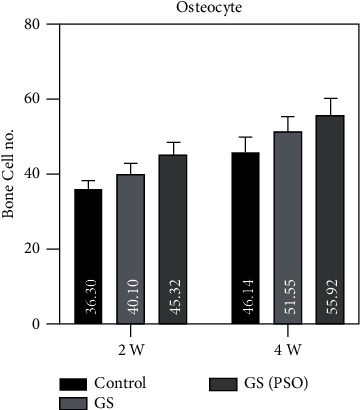
A bar chart representation of the mean and standard deviation of bone cells after 2- and 4-week time intervals for osteocyte, considering the C, GS, and GS/PSO groups.

Figures 7–9 show that OB and OCL mean values were higher at the 2-weeks duration, whereas OC mean values were higher at the 4-weeks duration in all studied groups.

A duration comparison for bone cells in all studied groups showed a highly significant result (*P* < 0.01) was obtained between 2- and 4-weeks regarding all bone cells by using the paired T test (Figures 7–9, Table 1).

**Figure 10:**
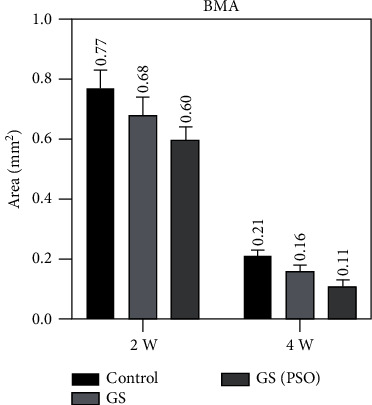
A bar chart representation of the mean and standard deviation of bone cells after 2- and 4-week time intervals, considering TA.

**Figure 11:**
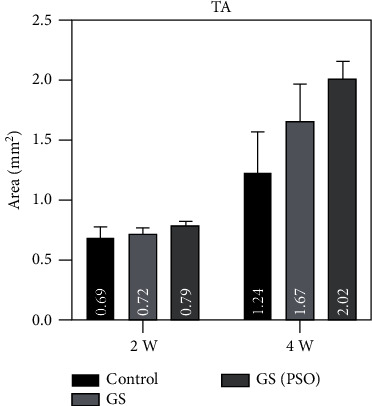
A bar chart representation of the mean and standard deviation of bone cells after 2- and 4-week time intervals, considering TN.

**Figure 12:**
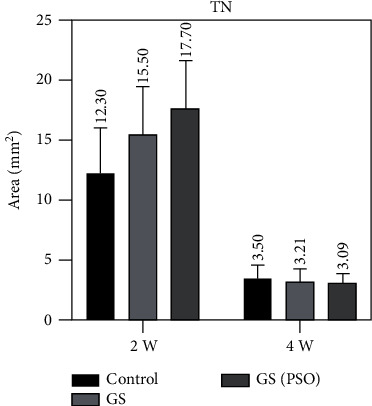
A bar chart representation of the mean and standard deviation of bone cells after 2- and 4-week time intervals, considering BMA.

Duration comparison for TN, TA, and BMA in all studied groups showed highly significant results (*P* < 0.01) obtained between 2- and 4-weeks duration by using paired *T* (Figures 10–12, Table 2).

Regarding multiple group differences for bone cells using the LSD test, the results showed highly significant (*P* < 0.01) and significant (*P* < 0.05) differences between all groups in both time periods (2- and 4-weeks) (Table 3).

Regarding multiple group differences for TN, TA, and BMA using the LSD test, the results showed highly significant (*P* < 0.01) and significant (*P* < 0.05) differences between all groups in both time periods (2- and 4-weeks) except for TN at 4 weeks (*P* > 0.05) (Table 4).

## 4. Discussion

The bone healing process is indicated by the deposition of bone matrix and the creation of trabecular bone, which decreases in number but grows in thickness throughout the healing process. The amount of newly formed bone formation in osseous defects is one of the most critical elements in determining the degree of bone regeneration in these defects [13]. In this study, different microscopical features were observed in the bone sections of different study groups, which were attributed to variations in the body's response to genetic or environmental factors, including the exogenous application of materials such as the absorbable gelatin sponge scaffold that contains bone-stimulating agents, causing new bone formation in the defect site [14].

The results of the histomorphometric study showed that the bone marrow space area decreased over time as the maturation and mineralization of newly formed trabeculae increased. This almost filled the defect sites, which could mean that the extract has a stimulating effect on osteoblastic bone formation.

The present study's histological findings demonstrated the creation of the bone trabeculae in the experimental and control groups, but at different rates of bone deposition and remodeling. Lacunae containing an OC were dispersed throughout the newly created trabeculae. OB bordered their trabeculae, enclosing the marrow tissue.

Most pomegranate seed oils usually contain unsaturated fatty acids in unconjugated form, except for a few that contain conjugated double, triple, or tetraene bonds. Examples of conjugated double and triple bonds are conjugated linoleic acids (CLA) and conjugated linolenic acids. The presence of conjugated fatty acids in oil has been the object of studies [15]. The pomegranate seed oil contains a higher concentration (>70%) of conjugated fatty acids [16].

Polyunsaturated fatty acids affect bone metabolism, which may be mediated through the regulation of osteoblastogenesis and OCL activity, change of membrane function, and a decrease in inflammatory cytokines [17].

Accordingly, this may explain the results of this study, where the combined application of GS and PSO at the defect site resulted in significantly more and thicker bone trabeculae than the other groups after 2-weeks. The number of OB in the GS/PSO group increased significantly compared with that in the C and GS groups. This could be attributed to the high flavonoid and punicic acid content of PSO, which is transformed to CLA according to the findings of Yogesh [18], who used an ethanolic extract of PSO to test the antiosteoporotic activity in ovariectomized (OVX) female albino rats and found a greater femoral length, weight, volume, and density than the control group. Increased OB proliferation and activity have been demonstrated to have a favorable effect on bone mineral density, as supported by a previous study by Spilmont et al. [19] who found that PSO intake significantly enhanced bone mineral density and avoided trabecular microarchitecture deterioration in OVX mice compared with OVX control animals. These findings are related to transcriptional alterations in bone tissue, indicating that osteoclastogenesis suppression and osteoblastogenesis improvement are involved in the process of bone formation.

Regarding the findings of this study, microscopical examination of histological sections 2 weeks after surgery revealed an increase in OB and OC and a decrease in OCL between the GS/PSO group and the GS group, with a significant increase in trabecular bone thickness and a decrease in BMA.

When comparing the OCL activity of the groups treated with GS/PSO with the C and GS groups after 2-weeks, we found that the GS group had a lower level of OCL activity. The number of OCL was lower in the GS/POS group, and there was a statistically significant difference when compared with other groups. This is in line with the findings of Zhang et al. [20], who found that feeding rats with modest doses of pomegranate seed oil improved the quality of the uterus, joint development, and density of the trabecular bone compared with feeding rats without pomegranate seed oil, suggesting that pomegranate seed oil has the ability to effectively suppress OCL activity.

The beneficial effects of flavonoids and isoflavone compounds on bone healing, including acceptable bone growth in the defect region, revealed that the progression of the bone healing process was accelerated after flavonoid application [21] without producing any complications such as infection or rejection. In addition to allowing for early OCL bioresorption, vascular invasion of macropores, and osteoblastic cell attachment on the surface of the graft at 14 days after implantation, progressive bone formation was observed at 2-weeks postoperatively. According to Kalli et al. [22], extracts rich in isoflavonoids may potentially be applied as natural antimicrobial agents.

Based on the results of this study, the increased trabecular bone area in the defect region treated with GS/PSO after 2-weeks of healing is attributable to an increase in osteoblastic activity and a decrease in OCL activity. This could be explained by the presence of flavonoids and punicic acid, which are found in different portions of pomegranate and contribute to improved bone production. A rise in the mean value of the OB number in bony defects may also be linked to the presence of these compounds in these defects. Moreover, the variations in the degree of the effects of different pomegranate extracts may be related to different constituents in the extracts, according to Monsefi et al. [23], who hypothesized that pomegranates can improve chondrogenesis and osteogenesis as they are a rich source of polyphenols, which can prevent bone loss.

Histologic examination of bone sections at 4-weeks showed mature bone tissue with numerous embedded OC and OB at the periphery in the C and GS groups. This is consistent with the findings of Elbahnasawy et al. [24], who discovered that greater regions of bone growth were detected with thick bony trabeculae after 4-weeks. Histological evaluation of the GS/PSO group indicated a mature bone with numerous regular arrangements of OC. According to Castelo-Branco and Cancelo Hidalgo [25], isoflavone compounds stimulate the transformation of mesenchymal cells into OB, stimulate bone formation in remodeling and repair processes, and are potent physiological inducers of OB differentiation and angiogenesis. In another study [26], a microscopic anatomy assessment of femur bone defects treated with recombinant isoflavone compounds revealed the presence of mature bone superimposed with active OB and filled with OC after 4-weeks. This supports the findings of the present study, where an increase in TA, a decrease in the OB mean value, and an increase in the OC mean number were observed in the GS/PSO group. In a previous study [27] to validate the in vitro osteogenic effects of PSO in primary calvarial OB cultures harvested from neonatal rats, PSO promoted the function of OB and played an important role in bone remodeling, which indicates that it may be an antiosteoporotic herbal candidate free from side effects and can be used as an alternative pharmacological agent for osteoporosis and skeletal tissues. Baban et al. [28] found that the oral supplementation of pomegranate extracts to rabbits can increase vitamin D levels, which play an essential role in bone mineralization in the case of bone defect creation, thus promoting the bone healing process.

## 5. Conclusions

According to the results obtained in this study and other studies, PSO is potentially effective in enhancing the bone healing process, particularly when it is combined with other biomaterials.

## Figures and Tables

**Table 1:** Duration comparison of bone cells in all studied groups.

Variable	Duration	C		GS		GS/POS	
		*T* Test	*P* Value	*T* Test	*P* Value	*T* Test	*P* Value
Osteoblast	Between 2- and 4-weeks	7.738	0.0001^*∗*^	6.637	0.0001^*∗*^	11.709	0.0001^*∗*^
Osteocyte	Between 2- and 4-weeks	1.629	0.138	−12.108	0.0001^*∗*^	−20.535	0.0001^*∗*^
Osteoclast	Between 2- and 4-weeks	7.477	0.0001^*∗*^	6.706	0.0001^*∗*^	5.622	0.0001^*∗*^

^*∗*^highly significant (*P* ≤ 0.001).

**Table 2:** Duration comparison of TN, TA, and BMA in all studied groups.

Variable	Duration	C		GS		GS/PSO	
		*T* Test	*P* Value	*T* Test	*P*Value	*T* Test	*P* Value
TN	Between 2- and 4-weeks	5.248	0.001^*∗*^	6.07	0.0001^*∗*^	5.209	0.001^*∗*^
TA	Between 2- and-4 weeks	−7.936	0.0001^*∗*^	−9.933	0.0001^*∗*^	−24.524	0.0001^*∗*^
BMA	Between 2- and 4-weeks	22.917	0.0001^*∗*^	26.936	0.0001^*∗*^	30.911	0.0001^*∗*^

^*∗*^High significant (*P* ≤ 0.001).

**Table 3:** Group comparison for bone cells at 2- and 4-weeks.

Variable	Duration	Groups	Comparison	
			Mean differences	*P* Value
Osteoblast	2-weeks	C/GS	−5.12	0.0001^*∗∗*^
		C/[GS/PSO]	−9.97	0.0001^*∗∗*^
		GS/[GS/PSO]	−4.86	0.0001^*∗∗*^
	4-weeks	C/GS	−4.34	0.0001^*∗∗*^
		C/[GS/PSO]	−8.46	0.0001^*∗∗*^
		GS/[GS/PSO]	−4.12	0.0001^*∗∗*^

Osteocyte	2-weeks	C/GS	−3.8	0.0001^*∗∗*^
		C/[GS/PSO]	−9.02	0.0001^*∗∗*^
		GS/[GS/PSO]	−5.22	0.0001^*∗∗*^
	4-weeks	C/GS	−5.41	0.005^*∗*^
		C/[GS/PSO]	−9.78	0.0001^*∗∗*^
		GS/[GS/PSO]	−4.37	0.0001^*∗∗*^

Osteoclast	2-weeks	C/GS	0.343	0.002^*∗*^
		C/[GS/PSO]	0.484	0.001^*∗∗*^
		GS/[GS/PSO]	0.241	0.002^*∗*^
	4-weeks	C/GS	0.09	0.030^*∗*^
		C/[GS/PSO]	0.19	0.0001^*∗∗*^
		GS/[GS/PSO]	0.1	0.032^*∗*^

^*∗*^significant (*P* > 0.05), ^*∗∗*^high significant (*P* ≤ 0.001).

**Table 4:** Group comparison for TN, TA, and BMA at 2- and 4-weeks.

Variable	Duration	Groups	Comparison	
			Mean differences	*P* Value
TN	2-weeks	C/GS	−3.200	0.01^*∗*^
		C/[GS/PSO]	−5.400	0.003^*∗*^
		GS/[GS/PSO]	−2.200	0.030^*∗*^
	4-weeks	C/GS	0.290	0.825
		C/[GS/PSO]	0.490	0.379
		GS/[GS/PSO]	0.120	0.508

TA	2-weeks	C/GS	−0.036	0.009^*∗*^
		C/[GS/PSO]	−0.104	0.02^*∗*^
		GS/[GS/PSO]	−0.068	0.080
	4-weeks	C/GS	−0.435	0.009^*∗*^
		C/[GS/PSO]	−0.189	0.0001^*∗∗*^
		GS/[GS/PSO]	−0.554	0.007^*∗*^

BMA	2-weeks	C/GS	0.092	0.002^*∗*^
		C/[GS/PSO]	0.175	0.0001^*∗∗*^
		GS/[GS/PSO]	0.087	0.002^*∗*^
	4-weeks	C/GS	0.043	0.0001^*∗∗*^
		C/[GS/PSO]	0.100	0.0001^*∗∗*^
		GS/[GS/PSO]	0.057	0.0001^*∗∗*^

^*∗*^significant (*P* > 0.05), ^*∗∗*^high significant (*P* ≤ 0.001).

## Data Availability

The data used to support the findings of this study are available from the corresponding author upon request.
